# Pituitary Pars Intermedia Dysfunction (PPID) in Horses

**DOI:** 10.3390/vetsci9100556

**Published:** 2022-10-10

**Authors:** Naomi C. Kirkwood, Kristopher J. Hughes, Allison J. Stewart

**Affiliations:** 1 School of Agricultural, Environmental and Veterinary Sciences, Charles Sturt University, Wagga Wagga, NSW 2678, Australia; 2 School of Veterinary Science, Gatton Campus, The University of Queensland, Gatton, QLD 4343, Australia

**Keywords:** endocrine, geriatric, hypertrichosis, immune dysfunction, laminitis, insulin dysregulation, α-melanocyte stimulating hormone (α-MSH), proopiomelanocortin (POMC) derived peptides, β-endorphin (β-END)

## Abstract

**Simple Summary:**

Pituitary pars intermedia dysfunction is the most common endocrine disease of geriatric horses and affects quality of life, immunocompetence and athletic performance. Clinical signs of pituitary pars intermedia dysfunction can include hypertrichosis (a long hair coat), muscle atrophy, a pendulous abdomen, recurrent infections, lethargy, lameness, polydipsia, and polyuria (drinking and urinating more than normal). Awareness of endocrine diseases such as pituitary pars intermedia dysfunction in the equine community has increased in the recent years, and new research is becoming available providing insight into prevalence of clinical signs, difficulties with current diagnostic techniques, and treatment of the condition. The purpose of this article is to review the current literature, to provide veterinarians access to current perspectives for pathophysiology, clinical signs, diagnosis, and treatment of pituitary pars intermedia dysfunction.

**Abstract:**

Substantial morbidity results from pituitary pars intermedia dysfunction (PPID) which is often underestimated by owners and veterinarians. Clinical signs, pathophysiology, diagnostic tests, and treatment protocols of this condition are reviewed. The importance of improved recognition of early clinical signs and diagnosis are highlighted, as initiation of treatment will result in improved quality of life. Future research should be targeted at improving the accuracy of the diagnosis of PPID, as basal adrenocorticotropic hormone (ACTH) concentration can lack sensitivity and thyrotropin releasing hormone (TRH) used to assess ACTH response to TRH stimulation is not commercially available as a sterile registered product in many countries. The relationship between PPID and insulin dysregulation and its association with laminitis, as well as additional management practices and long-term responses to treatment with pergolide also require further investigation.

## 1. Introduction

Pituitary pars intermedia dysfunction (PPID) is the most common endocrine disorder of geriatric horses, affecting 20–25% of horses over the age of 15 years [1,2,3,4]. Since the disease was first described in 1932 [5,6], considerable research has been conducted investigating PPID pathophysiology, prevalence of clinical signs, appropriate diagnostic techniques and treatment. In recent years, awareness of PPID among horse owners has grown [7], and veterinarians are increasingly testing for underlying endocrinopathies [8]. An increase in awareness has led to a substantial increase in research conducted in the field of equine endocrinology [9]. The purpose of this article is to review the current literature investigating anatomy of the equine pituitary gland, the pathophysiology, clinical signs, diagnosis, treatment and management of PPID, as well as directions for future research.

## 2. Anatomy and Physiology

**Figure 1:**
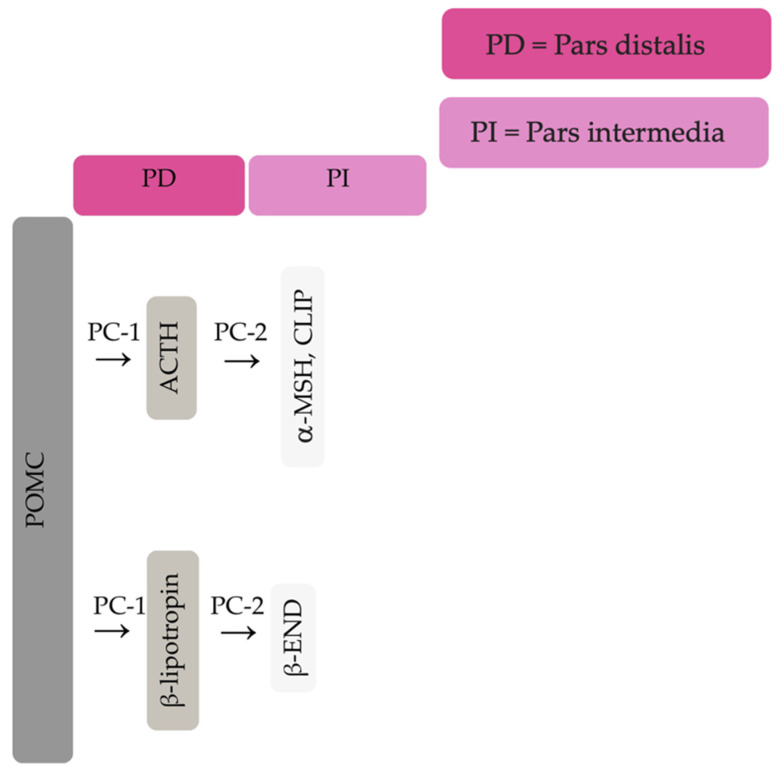
Simplified processing of POMC in the pars distalis by PC-1 to produce ACTH and β-lipotropin. Further PC-2 activity on ACTH and β-lipotropin to produce α-MSH, CLIP and β-END in the pars intermedia.

The equine pituitary gland is suspended by the infundibular stalk, ventral to the hypothalamus in the sella turcica [10]. The pituitary gland is composed of the adenohypophysis (pars distalis, pars intermedia and pars tuberalis), and the neurohypophysis (pars nervosa) [11]. In response to physiologic, pathologic or environmentally induced stressors, there is activation of the hypothalamic-pituitary-adrenal axis. This involves the paraventricular dopaminergic neurons of the hypothalamus synthesizing corticotropin-releasing hormone (CRH) which is released in the hypothalamic-hypophyseal portal vessels. There is local action of CRH on the corticotroph cells of the pars distalis, which leads to ACTH secretion. Adrenocorticotrophin acts on the zona fasiculata cells of the adrenal glands to synthesise and secrete glucocorticoids. The pars intermedia in horses is composed of melanotrope cells which are directly innervated by hypothalamic periventricular dopaminergic neurons which release dopamine. Dopamine acts on the D2 receptors of the melanotropes to inhibit cell proliferation and transcription of POMC [12,13,14]. POMC undergoes processing to produce ACTH, α-MSH, β-END and corticotropin like intermediate lobe peptide (CLIP) [4,15,16,17,18,19]. In the normal equine pituitary gland most ACTH is produced via enzymatic conversion of POMC by prohormone convertase I (PC-1) in the pars distalis [15]. ACTH is further cleaved by prohormone convertase 2 (PC-2) to produce α-MSH and CLIP [4,15,16,17,18,19]. β-END is produced via PC-2 conversion of β-lipotropin [20] (Figure 1). Melanotrophs are also stimulated by thyrotropin-releasing hormone (TRH) [21].

Closely linked to the hypothalamic-pituitary-adrenal axis is the hypothalamic-pituitary-thyroid axis [22]. Endogenous TRH produced by the hypothalamus causes an increase in thyroid stimulating hormone (TSH) produced by the pituitary gland, and an increase in circulating concentrations of thyroxine and triiodothyronine [23]. Glucocorticoids exert an inhibitory action on the hypothalamic-pituitary-thyroid axis by reducing TRH secretion by the hypothalamus [24,25]. Exogenous TRH administration will lead to binding of TRH receptors on the melanotrophs of the pars intermedia and secretion of POMC derived peptides [26].

Hypothalamic and pituitary secretions are physiologically influenced by several factors including stress, exercise, illness, photoperiod length, and climate [27,28,29,30]. In late summer and autumn, concentrations of α-MSH and ACTH in peripheral blood increase in normal horses, with more pronounced increases in animals with PPID [31]. These seasonal changes in ACTH and α-MSH concentrations require seasonally adjusted diagnostic cut-off values to diagnose PPID [2,32].

## 3. Pathophysiology of PPID

**Figure 2:**
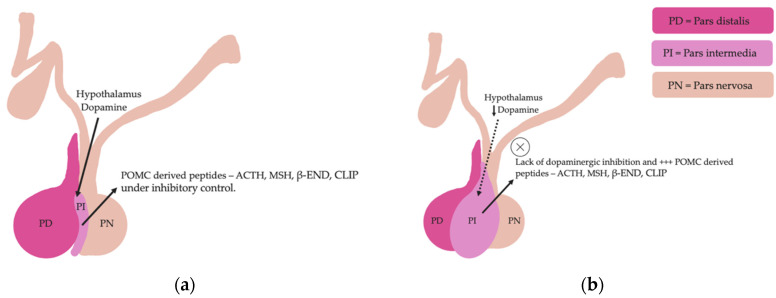
Normal equine pituitary gland (**a**). Equine pituitary gland affected by PPID demonstrating a loss of dopaminergic inhibition, and subsequent macroadenoma formation of the pituitary pars intermedia and overproduction of POMC derived peptides (**b**).

Pituitary pars intermedia dysfunction is one of the most common endocrinopathies in horses; however, the pathophysiology of PPID is incompletely understood [33,34,35,36,37,38]. The disease is caused by loss of dopaminergic inhibition of the pituitary PI due to oxidative-stress and subsequent neurodegeneration of dopaminergic neurons within the hypothalamus [17,39]. This theory was confirmed recently in a study that aimed to determine whether it was decreased dopamine or dopamine D2 receptor dysfunction that led to PPID. Tyrosine hydroxylase is a biomarker of dopaminergic neurons, and this retrospective study used 28 horses with PPID to measure tyrosine hydroxylase expression within the pars intermedia. A negative correlation between pituitary histomorphological grades and tyrosine hydroxylase was observed, confirming the involvement of reduced dopamine in the pathogenesis of PPID [38]. The loss of dopaminergic inhibition on the melanotropes of the pars intermedia results in hyperplasia, microadenoma or macroadenoma formation and overproduction of POMC derived peptides including ACTH and α-MSH (Figure 2) [16]. The ACTH secreted by the PI of horses with PPID is largely biologically inactive [17,40,41]. Subsequently, increased plasma ACTH concentrations do not result in adrenal stimulation and hypercortisolaemia [16,17,42,43,44]. Horses with PPID have resting serum concentrations of free thyroxine and TSH that are lower than age-matched control horses, with normal TSH responses to exogenous TRH administration [24]. Because glucocorticoids exert an inhibitory action on the hypothalamic-pituitary-thyroid axis [24,25], it has been hypothesized that increased glucocorticoid activity does exist, with prolonged tonic suppression of TRH [24].

Horses with PPID have increased concentrations of α-synuclein in dopaminergic nerve terminals within the pars intermedia compared to healthy geriatric horses [14,45]. α-synuclein extracted from the pituitary glands of PPID affected horses is misfolded and overexpressed compared to normal and age-matched horses [45]. In one study, the ultrastructure of α-synuclein from five each of young, aged and PPID affected horses was assessed using transmission electron microscopy and revealed no α-synuclein fibrils in young and aged horses. However, α-synuclein fibrils were detected in horses with PPID [45]. Accumulation of α-synuclein and oxidative stress can lead to cytotoxicity and subsequent degeneration of dopaminergic neurons in humans with Parkinson’s disease [46]. Further research is required to determine if misfolded α-synuclein is the cause or result of degeneration of dopaminergic neurons in horses with PPID [45].

## 4. Signalment

Twenty to twenty-five percent of horses over 15 years of age are affected by PPID [2,3,4]. There does not appear to be any sex predilection [3,4] or any breed predisposition to PPID [3]. Previously, despite the high prevalence of PPID in the geriatric equine population, owners were infrequently aware of the disease [4]. In 2012, free basal ACTH testing for PPID was well marketed, improving awareness of PPID in both owners and veterinarians [8]. However, despite recognising clinical signs, owners may be unlikely to seek veterinary advice [4]. Early clinical signs are also easily missed as they can be confused with normal age-related changes [4,47]. It is not recommended to test horses less than 10 years-of-age unless hypertrichosis is present [16] as younger horses are rarely affected by PPID [17]. The youngest horses reported with PPID were 7 years of age. This is considered unusual and early onset familial PPID was suspected [17].

## 5. Clinical Signs

**Figure 3:**
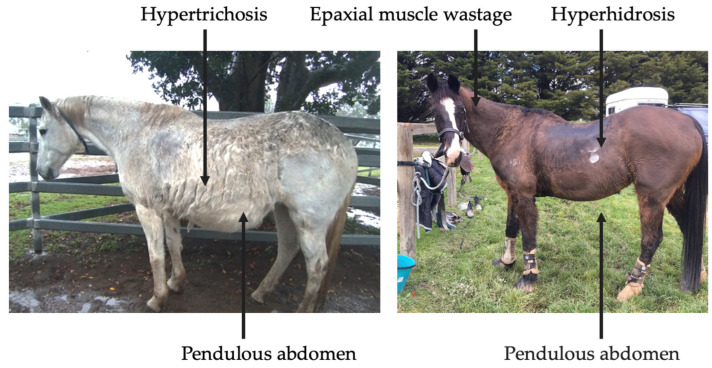
Clinical signs associated with severe, end-stage PPID.

Clinical abnormalities associated with PPID include hypertrichosis, lethargy, muscle atrophy, pendulous abdomen, polydipsia, polyuria, abnormal fat deposition, hyperhidrosis, recurrent infections, infertility, behavioural changes, insulin dysregulation (ID), and laminitis (Figure 3) [3,4]. However, mildly affected horses may only display subtle clinical signs, such as delayed shedding, which may not be easily identified [4]. Prompt recognition of clinical signs by owners and veterinarians may increase frequency of testing, leading to earlier diagnosis and initiation of treatment.

### 5.1. Hypertrichosis

Hypertrichosis is a commonly recognised clinical sign associated with PPID and is pathognomonic for the disease [4,48,49]. Hypertrichosis occurs due to an increased number of hair follicles in the anagen phase, or phase of active growth. However, the cause of hypertrichosis is poorly understood: theories include pressure of the hyperplastic pituitary gland on the thermoregulatory centre of the hypothalamus [49], or excess α-MSH produced by the pars intermedia [50,51].

Case series have been used to characterise the prevalence of clinical signs associated with PPID. However, many of these studies demonstrate selection bias as only horses with severe clinical disease, or horses from referral hospitals were included in the population making it difficult to extrapolate the results to all horses [52,53]. Unbiased sampling of over 300 horses determined that the prevalence of hypertrichosis was 33% as documented by the owner, and 41% as documented by the veterinarian [4]. A recent systematic review determined a prevalence of hypertrichosis of 69.9% in horses with PPID [3].

### 5.2. Laminitis

The cause of laminitis in horses with PPID is poorly understood. Laminitis can occur due to co-existing hyperinsulinemia [54,55]. Of 325 horses with PPID, 32% had basal, non-fasting hyperinsulinemia, and of those 66% had laminitis [4]. Laminitis has been experimentally induced via administration of insulin [56,57]. The hemidesmosome is a vital component of the basement membrane ultrastructure and hyperinsulinaemia can lead to reduced hemidesmosome density [58,59,60,61]. Reduced density results in widening, damage and weakening of the basement membrane and lamellar failure. The exact mechanism behind this ultrastructural pathology requires further investigation [58].

A multicentre study using a convenience sample of horses subjected to euthanasia found that all horses with PPID and laminitis had fasting hyperinsulinaeamia (median 74.1 μIU/L, IQR 49.9–349.5), whereas horses with PPID and without laminitis had serum insulin concentrations below 20 μIU/L [62]. Although the sample size in that study was small, hyperinsulinaemia was associated with endocrinopathic laminitis, but not necessarily with PPID [62]. A study of 10 non-PPID horses, 9 untreated PPID horses and 9 PPID horses undergoing treatment (pergolide) demonstrated that basal insulin and insulin responses to oral sugar tests (OST) did not differ suggesting that PPID and ID are likely distinct endocrine conditions [63]. Limitations of this study included small sample sizes and lack of randomisation of PPID horses into treatment groups. It is not known if, or how, PPID affects insulin concentrations [64,65]. Many studies have come to contradictory conclusions [62,66,67], but results of recent studies suggest that ID is an indicator of PPID chronicity, PPID and ID are different conditions or PPID exacerbates existing ID through undetermined processes [65,68,69,70,71]. More research is required to establish whether the conditions are different or interrelated.

Laminitis is the second most prevalent finding observed in horses and ponies with PPID and is often the first presenting complaint recognised by owners that seek veterinary attention [3,72]. A systematic review reported the prevalence of laminitis in the PPID population as 48.9% [3]. However, owner reported prevalence of laminitis in PPID affected horses was lower than the proportion of PPID affected horses that presented to veterinary clinics with hoof abnormalities indicative of chronic laminitis [3,73]. This finding could be due to increased concentration of serum β-END in horses with PPID, a potent endogenous opiate agonist, that provides analgesia and increases pain tolerance [7], or due to poor owner awareness [3]. In an unbiased Australian population, horses with PPID were found to be 4.7 (95% CI: 1.5–14.4) times more likely to have laminitis than control aged horses [4].

Typically, hoof abnormalities or lameness associated with laminitis are more common in pony breeds compared to horses [73], and lameness is less common in older horses with PPID compared to younger horses with PPID [47].

### 5.3. Muscle Wastage

Epaxial muscle wastage is common in horses with PPID [3]. Older horses with PPID appear more likely to demonstrate clinical signs of muscle wastage compared to younger horses [47]. Muscle wastage in horses with PPID is due to atrophy of types 2A and 2B muscle fibres [74]. However, it is unclear whether atrophy of muscle fibres is due to decreased protein synthesis, or increased proteolysis [75]. There are four mechanisms of proteolysis that contribute to protein degradation in horses: autophagy-lysosome, ubiquitin-proteasome, calcium-dependent calpains, and cysteine-protease caspase enzyme cascade [75,76]. Horses with PPID were found to have higher m-calpain than horses without PPID, suggesting that calpains may play a role in the development of PPID associated muscle wasting. However, other mechanisms of atrophy cannot be excluded, including posttranslational events that could interfere with protein activation, inactivation, and function [76]. Investigation of the involvement of the ubiquitin-proteasome pathway of proteolysis through the proteolytic marker muscle RING finger 1 (MuRF1), found no difference in MuRF1 or body phenylalanine kinetics between PPID affected and unaffected horses [77]. In contrast, other authors found MuRF1 to be more abundant in PPID horses compared to control horses, indicating involvement of the ubiquitin-proteasome pathway of proteolysis in PPID epaxial muscle wastage [75]. Differences in results among studies could be due to variations in population age, degree of muscle wastage, the muscle biopsied, time of sampling or evaluation at a translational or transcriptional level [76,77]. Identification of the pathophysiology behind epaxial muscle wastage in horses with PPID requires more research. Measurement of muscle wastage has not been consistent in the literature. Utilisation of a new muscle atrophy scoring system, once validated, may be useful [78].

### 5.4. Weight Loss

The pathophysiology behind weight loss in horses with PPID is unknown, and there are some horses that demonstrate a normal body condition or are obese [79,80]. The prevalence of weight loss in horses with PPID varies in the range of 5–88% between studies [3], and is well recognised by owners [4]. Older horses with PPID appear more likely to suffer from weight loss than younger horses with PPID [47]. As such, geriatric horses presenting to veterinary clinics with the presenting complaint of weight loss should be investigated for PPID.

### 5.5. Lethargy

It is difficult to objectively assess demeanour in horses, with the possibility of lethargy being confused with reluctance to move secondary to laminitis. Lethargy associated with PPID could occur due to increased β-END concentrations, as β-END is a natural potent opiate agonist [7,37,81]. Lethargy has been recognised in 4–95% of horses with PPID [3]. Older animals with PPID tend to be more predisposed to lethargy than younger horses with PPID [47].

### 5.6. Immune Dysfunction

Pituitary pars intermedia dysfunction has been associated with impaired immune function and an increased occurrence of opportunistic infections. In one study, opportunistic infections were found to occur in 35% of horses with PPID, compared to only 11% of aged horses that do not have PPID [7]. In humans and rodents, insulin, α-MSH, and other related melanocortins have been shown to reduce neutrophil function by reducing superoxide production (oxidative burst activity), migration and adhesion [82,83,84,85,86]: these alterations to neutrophil function, in addition to reduced chemotaxis, have also been demonstrated in PPID affected horses [87]. Impaired neutrophil function and reduced chemotaxis increases the risk of opportunistic infections [87].

Horses with PPID had a lower lymphocyte count, and decreased interferon γ production from peripheral blood mononuclear cells (PBMC) after stimulation with *Rhodococcus equi* and *Escherichia coli* and had increased interleukin-8 (IL-8) expression and transforming growth factor β expression compared to age-matched horses without PPID [88]. These findings suggest altered systemic immune function in horses with PPID through a reduced Th1 response [88]. Increased IL-8 gene expression in horses with PPID compared to age matched controls has been seen in other similar studies [89,90], particularly after TRH-stimulation testing, suggesting that alterations in IL-8 expression likely occurs as a result of PPID [90]. In humans, IL-8 contributes to neurodegeneration [91] and it is possible that IL-8 contributes to neurodegeneration in horses with PPID, but this requires further research.

One study evaluated the effect of PPID on immune function in horses through measurement of ACTH, insulin, total cortisol, free cortisol, complete blood counts, plasma myeloperoxidase (MPO) and cytokine/receptor gene expression in basal whole blood and in vitro stimulations of whole blood and PBMC [88]. No significant differences in resting insulin, total cortisol or free cortisol concentrations were identified between groups, reducing the likelihood of involvement of these hormones in immunosuppression [88]. White blood cells and lymphocyte counts were reduced in horses with PPID compared to age-matched controls. However, white blood cell and lymphocyte counts were still within normal limits in horses with PPID, even if significantly lower than healthy horses [88]. Plasma MPO concentrations in healthy horses and horses with PPID were not different, suggesting that the reduced neutrophil oxidative burst activity, migration and adhesion [82,83,84,85,86] does not occur as a result of altered MPO concentrations [88]. Horses with PPID and aged matched controls had similar responses to PMA/ionomycin stimulation, demonstrating that horses with PPID can respond adequately to non-antigen-specific stimuli. The immune dysfunction in horses with PPID in response to weaker stimuli may explain their higher risk of opportunistic infections specifically, rather than all pathogenic stimuli [88]. It is possible that immune responses are further reduced by ACTH. Adrenocorticotrophin has been shown to reduce immune function in other studies [92,93,94], and despite not increasing systemic cortisol concentrations in horses with PPID, could still be involved in immune dysfunction [88]. However, there was no difference in the immune function of horses with PPID that were treated with pergolide mesylate and untreated horses with PPID, suggesting that ACTH may have few immunomodulatory effects in horses [88]. Further research is required to determine the factors involved in immune dysfunction in horses with PPID.

In a study that compared healthy adult horses (10–16 years of age without PPID) and healthy geriatric horses (18–26 years of age without PPID) to determine the effects of ageing on immune function, geriatric horses demonstrated reduced IL-17α after PMA/ionomycin stimulation, increased tumour necrosis factor α (TNFα) production when stimulated with heat-inactivated *Rhodococcus equi,* reduced plasma MPO and reduced monocyte counts compared to adult horses [63]. The changes could represent a compensatory mechanism to ensure an adequate immune response in geriatric horses [63]. Other research has shown that aged horses show evidence of a pro-inflammatory state through an increase in pro-inflammatory cytokine gene expression and TNFα concentrations [89,90,95]. This phenomenon is known as inflamm-ageing and immunosenescence. In contrast, aged horses with PPID demonstrate inflammatory cytokines and interferon γ expression similar to middle-aged horses. It has been proposed that the increase in POMC anti-inflammatory hormones that occurs in horses with PPID counters age-related inflammation [89]. A more recent study comparing healthy adult controls, healthy geriatric horses, horses with PPID and horses with PPID and insulin dysregulation determined that PPID does moderately affect circulating markers of inflammation [90]. In contrast to other studies, there were limited differences in whole blood cytokine gene expression, serum cytokine concentrations or acute phase proteins between the groups [90]. However, there was a significant increase in IL-8 gene expression in horses with PPID and no ID compared to healthy geriatric horses. In that study, concurrent ID and PPID was associated with an increase in serum amyloid A (SAA) concentration [90], however, the sample size was only 10 horses with high levels of individual variability. Further studies on the phenomenon on inflamm-ageing in horses, as well as the relationship between PPID, insulin dysregulation and immunosuppression are warranted.

Compared to horses in similar environmental conditions, horses with PPID had higher faecal egg counts (eggs per gram (EPG)) before, and at 8, 10 and 12 weeks after ivermectin treatment, while age had no effect on EPG [96]. However, another study that used EPG as a marker of immune function failed to detect a difference in EPG between horses with pre-clinical PPID, healthy controls, and horses with pre-clinical PPID treated with pergolide. No horses included in the study had equine metabolic syndrome (EMS) or hyperinsulinemia, confirmed through an OST [97]. There are limitations of these studies, as EPG is not an accurate measure of immune function or size of parasite burden. Impacts of husbandry practices such as anthelmintic use and pasture management may have also influenced the EPG.

### 5.7. Polyuria and Polydipsia (PU/PD)

In one study, the prevalence of polyuria and polydipsia (PU/PD) was approximately 31% in horses with PPID [3]. However, PU/PD is likely underreported due to difficulty monitoring water intake and urine output in pasture-based situations. The cause of PU/PD is not well understood but could be explained by reduced antidiuretic hormone secretion from the pars nervosa due to compression by the PI [7,47,98]. This would explain why it is normally seen in more severe cases [47]. Although originally proposed, hypercortisolaemia antagonising antidiuretic hormone [99], and hyperglycaemia resulting in glucosuria and osmotic diuresis [100] are unlikely causes of PU/PD. Horses with PPID are not usually hypercortisolaemic [7,17,101], and are rarely hyperglycaemic or glucosuric [64,69]. Hyperhidrosis, fluid loss and resulting increased thirst have also been recently postulated to be involved in the mechanism behind PU/PD [98,102].

### 5.8. Hyperhidrosis and Anhidrosis

Warmer and more humid climates closer to the equator tend to have a higher frequency of hyperhidrosis and anhidrosis than cooler more temperate climates [47,103]. It is thought that hyperhidrosis occurs due to hypertrichosis in warmer climates. However, some horses continue to demonstrate this clinical sign even in cool climates or when whole-body clipped [7]. Anhidrosis results when sweat glands become exhausted [104]. Anhidrosis can result in exercise intolerance and even death as thermoregulation is impaired [103]. After intradermal terbutaline administration, plasma ACTH concentrations correlate more strongly to sweat production than α-MSH [37]. Unfortunately, the exact pathophysiology of hyperhidrosis and anhidrosis in horses with PPID has not been established.

### 5.9. Suspensory Ligament Degeneration

Suspensory ligament degeneration in aged horses, and horses with PPID is recognised. Recent studies have shown that in horses with PPID, there is reduced longitudinal arrangement of collagen fibres, inclusions of cartilage, haemorrhage and proteoglycan accumulation between fibres of the suspensory ligaments [105,106]. There are significantly more cells stained for glucocorticoid receptors in suspensory ligament samples of PPID horses compared to healthy horses. The investigators theorised that tissue disruption of cortisol metabolism may contribute to the suspensory ligament degeneration seen in horses with PPID [106].

### 5.10. Neurological Signs

Larger studies are required to better define and establish an accurate estimate of prevalence of neurological abnormalities in horses with PPID. Neurological signs are thought to occur because of a pituitary macroadenoma that can be diagnosed via computed tomography [107].

## 6. Diagnosis

A presumptive diagnosis of PPID can be made based on the presence of hypertrichosis in aged horses [108,109,110,111,112]. Laboratory testing is recommended in cases where treatment is financially feasible, where early or severe clinical disease is suspected [54], or to determine the response to treatment [54]. Currently, testing horses for subclinical PPID is not recommended [54]. However, the disease can have severe, life threatening consequences and ability to diagnose subclinical or mild PPID and initiate treatment before end-stage disease may be beneficial [40,47,113]. A recent prospective case series measured basal and TRH-stimulated ACTH concentrations in seven horses with subclinical PPID, and found that TRH-stimulation testing in late summer or early autumn (February and March in Australia) identified most horses that transitioned to clinical PPID over a 3.5 year period [113]. This was a small case series, so further research is required to determine if early diagnosis is warranted. Currently, there is no ante-mortem diagnostic test that can be completely relied upon for an accurate diagnosis of PPID [31,32]. Post-mortem evaluation of pituitary glands via histopathology has been used to confirm laboratory test results [111]. A grading system was created to determine the severity of pituitary pars intermedia histological changes [114], and the grade has been shown to correlate with laboratory results and clinical signs [114,115]. However, histopathology remains a subjective test, and concordance between pathologists is only moderate [116]. Pituitary size and histomorphology are also affected by season [115] and age [117] making the use of histopathology in diagnosis of PPID challenging [40]. Results of tests in horses that are affected by early or subclinical disease, are often equivocal [113]. Untreated PPID cases due to false-negative test results can lead to morbidity through muscle wastage, opportunistic infections, and difficulty thermoregulating. False positive test results may lead to unnecessary treatment. This is not only a financial burden, but the risks associated with long-term treatment with pergolide have not been established [2,118]. It is imperative that diagnostic tests be interpreted with care to reduce the incidence of false positive and false negative results [32].

Current recommendations for diagnosis of PPID are to measure the basal ACTH concentration in horses with obvious clinical signs, utilising seasonally adjusted diagnostic thresholds and an equivocal zone [54]. For the diagnosis of early disease, measurement of TRH-stimulated ACTH concentrations is recommended [2,54]. When finances or availability of laboratory testing of ACTH concentrations are not available, response to therapy with pergolide mesylate may be used as a more practical approach to diagnosing PPID in aged horses with generalised hypertrichosis [54]. Other diagnostic techniques include measurement of endogenous α-MSH and the now superseded overnight dexamethasone suppression test. Measurement of endogenous α-MSH, it is not commercially available and did not perform as well as basal ACTH [28,31,119]. Seasonally adjusted DCOV are yet to be established for the overnight dexamethasone suppression test. Although not discussed in this review, additional diagnostic testing for hyperinsulinemia is recommended for horses with regional adiposity and/or a history of laminitis, as management practices are altered in these horses [120].

### 6.1. Basal ACTH

Basal ACTH is the most popular and common test used for diagnosis of PPID as it is easy to perform and requires only a single blood measurement [121]. Interpretation of results must take into consideration stress, illness, pain, season, age, sex, and body condition score, as all these factors can increase concentrations of ACTH [30]. Breed differences in ACTH concentration have been observed with a greater and prolonged peak in ACTH concentrations in late summer and autumn in Shetland and Welsh ponies compared to Warmbloods, Thoroughbreds and Cob breeds. These differences were not observed outside of late summer and autumn. Arabians and donkeys had ACTH concentrations greater than other breeds for a more prolonged period from May to November [122,123]. These differences in ACTH concentration do not appear to have led to overrepresentation of these breeds in diagnosis of PPID [3]. The test can be performed at any time of day [124,125]. Performing paired measurement of ACTH has no benefit to performing a single measurement [125]. If these results are equivocal, a TRH stimulation test should be performed due to the greater accuracy of this test [2,40].

The blood sample should be collected in an EDTA tube, centrifuged, kept at 4 °C and analysed as soon as possible after sampling, ideally within 24 h [126,127]. If the sample cannot be tested immediately, the centrifuged plasma should be frozen as heat results in degradation of ACTH [128]. Multiple freeze-thaw cycles should be avoided as this will alter the ACTH concentration [129].

In all horses, basal ACTH follows a circannual rhythm that demonstrates a peak in concentration in the autumn months. Basal ACTH was thought to be the most sensitive and specific diagnostic test in the autumn due to increased magnitude of differences in ACTH concentrations between affected and unaffected horses [31]. However, more recent literature has demonstrated the accuracy of basal ACTH is reduced in March (September in the Northern hemisphere). Basal ACTH concentration still had good accuracy for diagnosis of PPID in March (accuracy 0.8–0.9), but accuracy was improved to excellent (accuracy > 0.9) in April and May [2]. Meta-analyses and systematic reviews have been performed to establish the sensitivity and specificity of basal ACTH. Although not broken down by season, the median sensitivity and specificity of basal ACTH in one study were 75.5% and 95.2% respectively [36], while another study reported the mean sensitivity and specificity at 66% and 87% respectively [130]. Based on these results, basal ACTH is excellent for ruling out PPID, and less accurate for detecting the condition unless the horse has severe clinical signs. As such, basal ACTH is not recommended for screening purposes or use in horses with early disease [130]. However, both studies identified biases, between-study variations and suboptimal study designs and populations. Less biased studies examining the diagnostic accuracy of basal ACTH are required to accurately establish sensitivity and specificity.

Monthly reference intervals (RI) and DCOV for the interpretation of ACTH results were established in a study of 106 mature horses. In this study DCOV were recommended to improve the detection of early PPID, and RI were recommended to reduce the likelihood of false positives [2]. This study proposed that determination of basal ACTH concentrations is accurate for diagnosis of PPID in horses, providing these DCOV and RI are used, and the results are interpreted within the clinical context. The TRH stimulation test may improve the accuracy of diagnosis of PPID irrespective of season [2]. Another recent retrospective study using a population of 75,892 horses used an indirect approach to calculate diagnostic thresholds. This study similarly proposed that diagnostic thresholds should be used alongside clinical judgement, and the appropriate threshold should be chosen when there is a reason to avoid false positive or false negative results [32]. This study established diagnostic thresholds using intervals as short as one week. Inter-weekly variability was low for most of the year, with a nadir in June to December (northern hemisphere) [32]. The Equine Endocrinology Group recommends using seasonal thresholds with equivocal zones for interpretation of results. Prior to recommending treatment for horses without strong clinical signs that have basal ACTH results that fall within equivocal zones, re-testing or use of a TRH stimulation test is recommended [54].

### 6.2. Thyrotropin-Releasing Hormone (TRH) Stimulation Test

In early PPID or when clinical signs are mild, the TRH stimulation test is useful for increased accuracy of diagnosis [1,2,40,113,121]. Plasma ACTH is measured before and either 10 or 30 min after administration of 1 mg TRH IV (0.5 mg for ponies). Thyrotropin-releasing hormone increases ACTH and α-MSH concentrations in plasma through stimulation of TRH R1 receptors on the pituitary pars intermedia and pars distalis [1,21,40,119,131]. Thyrotropin-releasing hormone stimulation in normal horses results in POMC derived peptide secretion by the pars distalis that is restricted by glucocorticoid negative feedback. In horses with PPID, TRH stimulation results in POMC-derived peptide secretion by the melanotrophs of the pars intermedia, and excessive ACTH secretion by hyperplastic or neoplastic melanotrophs that is unaffected by a negative feedback loop, resulting in a dramatic increase in ACTH concentration [1,2,21,26]. As with basal ACTH, the TRH stimulation test should be interpreted in accordance with seasonally adjusted DCOV to improve diagnostic accuracy [2,54]. Several investigators recommend not using the TRH stimulation test in the autumn [40,42,54], and repeatability of the test in autumn appears to be reduced [132]. However, recent studies have found that while variation in ACTH concentrations are greater in autumn, it should not impact diagnostic accuracy providing DCOV and RI are used [2,133]. The disparity in results could be due to the difference in ages of the populations investigated, as well as sample sizes.

The TRH stimulation test has been associated with side effects such as yawning, muscle fasciculation, transient coughing, lip-smacking and the flehmen response, but appears safe. The test is applicable for both hospital and ambulatory practice [40]. Measuring ACTH concentration 10-min post TRH administration is favoured by ambulatory practitioners for time efficiency, hence its recommendation by the Equine Endocrinology Group (EEG) [54]. However, as there is a much larger variation in the ACTH concentration 1 min pre and post the 10 min time point [40,42,131,134], compared to 1 min pre and post the 30 min time point [2,132] (where the ACTH response curve is flatter), some researchers favour using the 30 min time point due to greater accuracy when repeated samples are required [134]. Further research is required to determine the variability of ACTH concentrations 30 min post TRH administration [134]. Prior to the availability of a compounded product in the USA, most research papers utilise the chemical grade TRH (Sigma-Aldrich Pty Ltd. (subsidiary of Merck), North Ryde BC, Australia or synthetic thyrotropin releasing hormone, Sigma-Aldrich Inc, St. Louis, MO, USA) [2,121,124,127,129,132]. Although TRH is now available as a compounded product in the USA, in Europe and Australia TRH is not available as a sterile registered product, and use tends to be limited to referral hospitals [132,133,134]. In the UK and most of Europe, the use of unlicenced chemicals is restricted, limiting the use of TRH for the diagnosis of PPID.

### 6.3. Dexamethasone Suppression Test

The dexamethasone suppression test for detection of PPID requires two consecutive visits making it a less popular option. It is also less valuable in the identification of early disease, and administration of dexamethasone has been associated with negative consequences such as laminitis [135,136]. Serum cortisol concentration is measured before and 18–20 h after administration of 0.04 mg/kg dexamethasone IM [28,137,138]. In normal horses, administration of dexamethasone results in negative feedback on the corticotropes of the pars distalis, suppressed ACTH secretion and a reduction in plasma cortisol concentration. However, in horses with PPID, cortisol secretion is maintained as the melanotropes of the pars intermedia continue to secrete large quantities of ACTH [1]. Results of the dexamethasone suppression test are frequently inconsistent with TRH stimulation test results [40]. There is also some evidence to suggest that season influences the result of the dexamethasone suppression test, with false positives more likely in the autumn months [27,139]. The dexamethasone suppression test is no longer recommended if laboratory measurement of ACTH concentration is available.

### 6.4. Plasma α-MSH

Unlike ACTH, α-MSH is a direct product of the pars intermedia [21]. In horses with PPID, the hyperplastic pars intermedia secretes α-MSH in quantities in excess of ACTH production [21,119,131], indicating that α-MSH may increase earlier in the disease process [5,51]. Measuring α-MSH has shown to have improved diagnostic accuracy than basal ACTH and may be more useful in detecting early disease [21,28,51,119,131]. α-MSH has a higher specificity and sensitivity than ACTH in the autumn months, and specificity is also greater than that of basal ACTH in the non-autumn months, but the sensitivity reduces [31]. Measurement of α-MSH has been identified as a test that could be useful in the diagnosis of PPID, as unlike ACTH, α-MSH is not known to be confounded by variables such as stress, transportation, exercise and pain [2,21]. Similar to ACTH, α-MSH concentration is influenced by season and is highest in the autumn months [21,28,51,119]. Seasonally adjusted DCOV for basal α-MSH were established using the Youden index and compared to ACTH DCOV, and found that α-MSH and ACTH were highly correlated [31]. α-MSH is not as stable as ACTH in chilled whole blood, remaining stable for 8 h prior to centrifugation, plasma separation and freezing [51]. Therefore, its use may be restricted in ambulatory practice. Laboratory measurement of α-MSH is not commercially available. Some research has been established investigating α-MSH response to TRH administration, but it is not any more useful than measuring ACTH after TRH administration [131]. Increases in α-MSH in response to TRH were substantially greater than ACTH in the autumn months [21,119], which may complicate the use of the test unless seasonally adjusted DCOVs are established. However, this may also prove to be a valuable diagnostic test if interpreted correctly [119].

### 6.5. Imaging

Computed tomography (CT) [107] and more recently, magnetic resonance imaging (MRI) [140] have been used to evaluate the equine pituitary gland, and both modalities appear to provide adequate detail [107,140]. Because no laboratory test for PPID can be completely relied upon, imaging may be useful for earlier detection of the disease [140]. Imaging may also be useful to assess pituitary size, surrounding structures, and obtain a more accurate prognosis [141]. The use of imaging modalities is limited at this stage due to expense and accessibility, but may become a suitable diagnostic technique in the future [107].

## 7. Treatment and Management

Pituitary pars intermedia dysfunction can be managed effectively with pergolide mesylate and husbandry practices [118]. Medical treatment of PPID will is unlikely to resolve the underlying pituitary pathology but will control most of the clinical signs [107]. Horses with PPID may benefit from aggressive preventive health care such as parasite control to minimise worm burdens [96], regular dentistry, and corrective farriery for management of laminitis [5,7]. Appropriate treatment will improve the horse’s quality of life.

### 7.1. Pergolide Mesylate

Pergolide is the treatment of choice for PPID and is the only drug registered for use in horses with PPID [1,142]. Pergolide is a synthetic, ergot-derived D2-dopamine full agonist that acts with greatest affinity on the inhibitory D2 receptors expressed on the melanotrophs of the pars intermedia. Pergolide is also a full agonist at D3 receptors and has some activity at D1 receptors [142]. Activation of the D2 receptor reduces POMC production by the pars intermedia within hours, resulting in reduced severity of clinical signs and limiting sequelae that accompany PPID [17,142,143]. Pergolide provides an exogenous supply of dopamine that would usually be provided by the hypothalamus and restores pituitary pars intermedia function through the provision of dopaminergic inhibition [121,142,144]. Restoration of dopamine has been observed to reduce ACTH concentrations to within reference intervals in 28–74% of cases [118].

**Figure 4:**
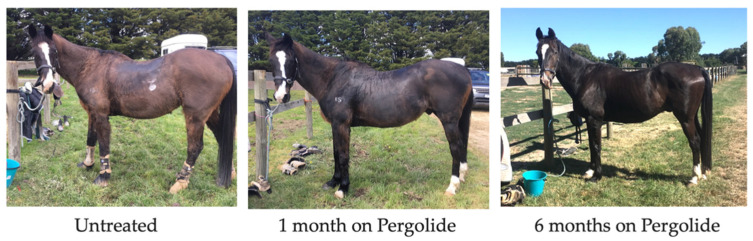
18-year-old Irish Sport Horse clipped coat untreated; after one month of pergolide, and after six months of pergolide.

**Figure 5:**
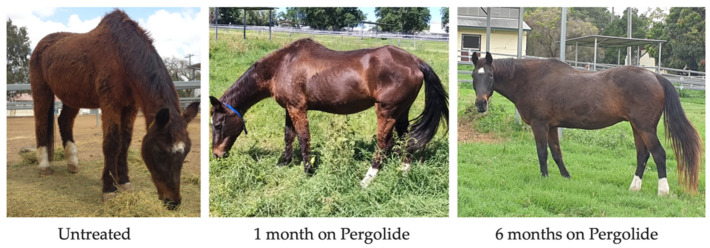
25-year-old Australian Stockhorse mare untreated; after one month of pergolide and six months of pergolide.

There is little evidence regarding the long-term efficacy of Pergolide for improving prognosis [145]. However, pergolide use was associated with increased short-term survival in a recent retrospective study [47], and also a reduction in clinical signs in 40–100% of cases in a recent systematic review [118]. Hypertrichosis score is reduced in horses administered pergolide (Figure 4 and Figure 5) [37,80,146], with the number of hair follicles in the anagen phase in PPID horses reduced to levels consistent with horses unaffected by PPID [49,75]. In a systematic review, hypertrichosis improvement ranged from 30–100%, with improvements in hyperhidrosis by 15–45%, lethargy/poor performance by 20–47%, abnormal fat distribution by 0–33% and laminitis by 32–75% [118]. Pergolide has no effect on insulin concentration [75] or immune function [88]. Markers of proteolysis (MrRF1) and muscle atrophy were also not altered by pergolide treatment [75]. Conversely, in a clinical trial that used a subjective muscle atrophy score, it was found that by day 180 of treatment, pergolide reduced muscle atrophy in 46% of horses [147]. However, this trial did not utilise objective measures of muscle atrophy, and as no control group was used it is difficult to draw conclusions. Use of the new muscle atrophy scoring system (once validated) may assist in objective measurement of muscle atrophy and response to treatment [78]. Evaluation of a clinical response is frequently subjective. Further research is required regarding the efficacy of pergolide with regard to specific clinical signs [118]. Pituitary hyperplasia/ macro or microadenoma size may not reduce with pergolide treatment [107]. In one study, 6/6 horses with PPID treated with pergolide saw an improvement in one or more clinical signs after six months on treatment. However, pituitary gland length as measured using CT increased. The second pituitary measurements were taken in the autumn months when there is an increase in ACTH secretion in all horses, which could also explain the lack of reduction in pituitary size [107]. Neurological signs resulting from the space occupying lesion may not improve with pergolide treatment if the pituitary size does not decrease. However, further research is required to determine the impact of pergolide on pituitary hyperplasia and adenoma size.

It is recommended to commence dosing pergolide at 0.002 mg/kg (1 mg for a 500 kg horse) once a day orally [148]. From a study investigating the pharmacokinetics and pharmacodynamics of pergolide, it was established that oral pergolide supresses the activity of POMC production in the pars intermedia within hours. It was also determined that both ACTH and pergolide concentrations fluctuate, consistent with a terminal elimination half-life of less than 12 h [142]. Because of this, twice daily dosing of pergolide could be beneficial, contrary to current recommendations. Further research would be beneficial to confirm this finding. Once the initial dose of pergolide has been commenced, it is recommended that basal ACTH concentration be reassessed and clinical signs re-evaluated one month after initiation of treatment to establish response to therapy [1]. The dose can then be titrated to effect at increments of 0.001 mg/kg and reassessed monthly to establish an appropriate dose [1,7]. The time to effect of pergolide is rapid, and there may be merit in assessing ACTH concentrations days to weeks after initiation of treatment as opposed to one month after treatment [142]. Assessing response to treatment sooner after initiation will allow the appropriate dose to be established earlier, and potentially reduce morbidities associated with PPID if the dose is shown to be inadequate. Recheck examinations should be performed every 6–12 months once an appropriate dose has been established, as the disease can continue to progress, and horses can develop drug tolerance [7]. Treatment should be continued for the duration of the horse’s life. Monitoring of both endocrine dysfunction and clinical response to treatment is essential for effective control of PPID.

Pergolide is a prohibited substance under several equestrian federations and subsequently horses are unable to compete unless the drug is withheld several days prior to competition [143]. Pergolide is commercially available in a tablet form throughout most of the world (Prascend™ 1 mg Boehringer Ingelheim). It is also available as an approved liquid formulation in Australia (Ranvet’s Pergolide 1 mg/5 mL Ranvet) and is commonly compounded in a liquid form. Where possible, the tablet form should be recommended, as the liquid is unstable in the light, should be refrigerated and has a short shelf-life of only one month [149].

Side effects from pergolide include inappetence, colic, sweating, diarrhoea and neurological signs [5,142]. If side effects are present, the dose should be reduced, and gradually increased over a period of weeks to the appropriate efficacious dose [5,7].

Research investigating the use of pergolide in the treatment of Parkinson’s disease in humans has established that pergolide also protects dopaminergic neurons under conditions of elevated oxidative stress [7,150]. This may reduce the progression or development of disease. Early detection and treatment of PPID should therefore be encouraged. Further research is required to determine the benefits of beginning pergolide therapy early in the course of disease in terms of reduction in morbidity and long-term prognosis.

Other medical therapies of PPID include the serotonin antagonist cyproheptadine [80,109,146,151,152], the 3-hydroxysteroid dehydrogenase inhibitor trilostane [64,153,154], and adrenolytic mitotane [155,156]. However, there is currently limited evidence to suggest their efficacy in the treatment of disease [6,64,146,152,154,155,157].

### 7.2. Husbandry Practices

Horses with PPID require excellent management and preventive healthcare [5,7]. A potential link has been established between PPID and hyperinsulinaemia that can lead to laminitis [62,65,158]. While ACTH concentrations are not predictive of laminitis risk [159] and hyperinsulinaemia and PPID may not necessarily be linked [62], if horses with PPID have a history of laminitis or have been diagnosed with insulin dysregulation, feeds high in non-structural carbohydrates should be avoided [71,159]. However, because horses with PPID often experience weight loss and muscle wastage, close attention should be paid to the horse’s body condition score. If horses begin to lose weight, supplementary feeding should be implemented to improve body condition and the diet should be tailored based on insulin dysregulation status. All diets should be tailored to age, body condition and athletic use [71]. A strong negative association between serum vitamin B12 and PPID was observed when investigating the relationships of inflamm-ageing with nutrient levels and PPID in a population of 42 senior horses [160]. Vitamin B12 deficiency has been associated with Parkinson’s disease [161], Alzheimer’s disease [162] and human patients with Cushing’s disease [163], and is essential for normal central nervous system function [164,165]. There may be a correlation between low vitamin B12 and PPID in aged horses, and supplementation of vitamin B12 may be warranted [71]; however, this requires further research.

Immune function is altered in horses with PPID and subsequently strict preventive healthcare measures and biosecurity protocols should be put into place [63]. Regular dental examinations and faecal egg counts should be performed, due to an increased risk of age-related dental disease [71] and the increased risk of secondary infections [7,63] and endoparasitism [96]. Horses with dental disease that cannot tolerate consumption of long fibre length dry matter should be offered chaff or fibre pellets (beet pulp) to meet dry matter intake requirements [166]. Horses with difficulty thermoregulating due to anhidrosis or hypertrichosis should be provided with ample shade, fresh water and body clipping as required [7].

Recent studies have investigated alternative therapies to reduce hypertrichosis in horses with PPID. The hormone responsible for the regulation of hair growth in horses is prolactin, which is supressed by melatonin. Blue light therapy has been shown to suppress melatonin, and subsequently increase prolactin concentrations in non-PPID horses, leading to lighter hair weights in these horses [167]. Interestingly, blue light therapy in the form of an Equilume Curragh light mask (Equilume Ltd., Kildare, Ireland) used in horses affected by PPID also resulted in lighter hair weights and reduced severity of hypertrichosis compared to control PPID horses. However, this had no effect on plasma ACTH concentrations suggesting that ACTH cannot be regulated via the manipulation of melatonin, and that blue light therapy cannot be used as a sole treatment for PPID [168]. It must be noted that in this study, shedding score observations may not be meaningfully evaluated statistically due to a small population and inability to randomise the control and treatment groups. Veterinarians were also not blinded to the horse’s group allocation which may have led to detection bias in results. Large-scale research with randomisation and blinding is recommended for future studies to better facilitate statistical analysis and reduce bias.

## 8. Conclusions

Pituitary pars intermedia dysfunction is a common disease of aged horses that results from loss of dopaminergic inhibition of the pars intermedia. Early clinical signs of PPID are frequently missed, and the disease is often undiagnosed until the condition is advanced. The most accurate diagnostic test for identification of early disease is the TRH-stimulation test, and the most common diagnostic test is basal ACTH concentration. Both tests should be interpreted in accordance with appropriate DCOV, RI or seasonal thresholds, depending on the clinical presentation and need to reduce false negative or false positive results. Diagnosis based on clinical signs alone is possible, though once clinical signs are obvious, the disease could be severe [113]. To increase quality of life and athletic performance, treatment with pergolide mesylate is recommended. Improved recognition of early clinical signs may result in earlier initiation of treatment and improved quality of life.

## 9. Future Directions

Research investigating the relationship between laminitis and PPID with and without hyperinsulinemia is warranted, as the relationship is still incompletely understood. A study investigating research priorities found that owners were concerned about management strategies (feed and turnout) for horses with PPID, and the response of PPID horses with laminitis to treatment with pergolide [145]. Investigating diagnostic accuracy of testing procedures in horses with subtle clinical signs may result in earlier diagnosis and initiation of treatment which could also improve survival [47]. A largescale randomised placebo controlled, double blinded study with pergolide and placebo treating horses with early and obvious PPID would be required to determine the true effects of pergolide on longevity and quality of life, and if earlier intervention with pergolide is warranted.

## Data Availability

Not applicable.
